# “Now You Have Become Doctors”: Lady Health Workers' Experiences Implementing an mHealth Application in Rural Pakistan

**DOI:** 10.3389/fgwh.2021.645705

**Published:** 2021-05-14

**Authors:** Mai-Lei Woo Kinshella, Sana Sheikh, Sohail Bawani, Michelle La, Sumedha Sharma, Marianne Vidler, Laura A. Magee, Peter von Dadelszen, Zulfiqar Bhutta, Rahat Najam Qureshi, Beth A. Payne, The CLIP Working Group

**Affiliations:** ^1^Department of Obstetrics and Gynaecology, University of British Columbia, Vancouver, BC, Canada; ^2^Department of Obstetrics and Gynaecology, Aga Khan University, Karachi, Pakistan; ^3^University of British Columbia, Vancouver, BC, Canada; ^4^Department of Women and Children's Health, King's College London, London, United Kingdom; ^5^Centre for Global Child Health, SickKids Hospital, Toronto, ON, Canada; ^6^Center of Excellence in Women and Child Health, Aga Khan University, Karachi, Pakistan

**Keywords:** PIERS on the Move, mHealth, community health workers, lady health workers, Pakistan

## Abstract

**Background:** PIERS on the Move (POM) is a mobile health application developed to support community health workers identification and management of women at risk of adverse outcomes from pre-eclampsia. The objective of this study was to evaluate the impact of using POM in Pakistan on Lady Health Workers' (LHWs) knowledge and self-efficacy related to caring for women with pre-eclampsia, and their perception of usefulness of the tool.

**Methodology:** An evaluation was designed for health care workers involved in the Community-Level Intervention for Pre-eclampsia (CLIP) cluster randomized trial from 2014 to 2016 in Sindh Province, Pakistan (NCT01911494). A semi-structured focus group guide was developed based on the Technology Acceptance Model (TAM), which theorizes that an individual's behavioral intention to use a system is determined by perceived usefulness and ease of use. Preliminary qualitative analysis was undertaken by the Pakistan and Canadian teams to create a coding framework for full qualitative analysis, which was completed using NVivo12.

**Results:** Three key informant interviews were conducted with two Lady Health Supervisors and one Senior Medical Officer. Sixty-two LHWs were included in three focus group discussions. LHWs found the POM app easy to use and useful for their work as a helpful repository for maternal health information and guiding counseling and management of pre-eclampsia. LHWs reported increased knowledge and confidence in their work. Availability of clinical homecare, including postpartum, was felt to positively impact healthcare provided to pregnant and postpartum women. Potential community level impacts included strengthening relationships between health care providers and communities and between LHWs and the health system. LHWs shared reports of earlier care-seeking and increased awareness of maternal health issues by community members.

**Conclusions:** LHWs carry a large burden of responsibility for community health in rural Pakistan and appreciated the investment in their skills and capacity during the CLIP trial with the POM app. Investing in communications technology for community health workers through improved referrals and follow up may strengthen cohesiveness of the health system overall.

## Introduction

Health infrastructure gaps and shortages of qualified health personnel hinder delivery of timely antenatal care in low- and middle-income countries (LMICs), particularly at rural areas (1, 2). Mobile technologies for health (mHealth), through supporting clinical task-sharing between cadres of health workers, have the potential to reduce the time, distance and costs for health care delivery in these settings. Thereby, mHealth may overcome issues of inadequate financing, poor access to information, limited human resources, and challenges of geographic access to care for women with pre-eclampsia in LMICs (3–5).

Increasing evidence shows that mHealth interventions can improve antenatal and postnatal care attendance, facility based deliveries, skilled attendance at birth and vaccination rates (3, 6) and enables more efficient data reporting (1). However, while there has been widespread excitement about the importance and potential benefits of mHealth, realizing these benefits is challenged by difficulties around implementation (5) and especially around evaluating the needs for health care workers to effectively use digital health (4). A review found that a lack of interaction between health care workers and mHealth system developers was a key gap in the mHealth process (1).

One of three “Community-Level Intervention for Pre-eclampsia (CLIP)” Trials was completed in Pakistan from 2014 to 2016 (NCT01911494). The aim of the trial was to reduce maternal, perinatal and neonatal mortality, and maternal and neonatal severe morbidity by 20% through providing a pre-eclampsia focused package of care. The prevalence of pregnancy hypertension is 9.3% (7) in Sindh province, where the study was conducted. Pre-eclampsia, the most dangerous of the hypertensive disorders of pregnancy, is the third leading cause of maternal deaths in Pakistan, contributing an estimated 11% of all the country's maternal deaths (8). Pre-eclampsia is a disorder of pregnancy, typically defined by new development of hypertension and proteinuria or other adverse conditions reflecting systemic endothelial dysfunction after 20 weeks of pregnancy (9). The CLIP intervention was a complex package of clinical and non-clinical activities. Non-clinical intervention activities included community engagement sessions with male stakeholders and education sessions with pregnant women and their families in their home. The clinical intervention was delivered at pregnant women's homes by LHWs, who are a cadre of lay health workers deployed by the government in Pakistan (10). LHWs learned new clinical tasks for the trial and provided home-based pregnancy care guided by the PIERS on the Move (POM) mHealth application accessed via a tablet. LHWs were trained by CLIP staff to use the POM app, measure blood pressure (BP), proteinuria, oxygen saturation (all through point of care devices integrated with the POM mobile device), and to administer drugs to pregnant women, when indicated.

The objective of this study was to evaluate the impact of using the POM app on Lady Health Workers' knowledge and self-efficacy related to caring for pregnant women, and their perceptions of usefulness, ease of use and community reception. As part of this, we evaluated LHWs' perception of strengths and weaknesses of the tool's current design and documented recommendations for app improvements.

## Methodology

### Details of the CLIP Intervention Being Evaluated

POM was developed to support community health workers (CHWs) in each of three countries (i.e., Pakistan, India, and Mozambique) to identify and manage women at risk of adverse outcomes from pre-eclampsia. The POM app integrates the miniPIERS model (11), which uses demographics, clinical symptoms and signs to calculate maternal risk of developing a complication of pre-eclampsia, with added accuracy by phone oximetry (measures oxygen saturation) (12). The POM app guides CHWs, such as LHWs, through the process of assessment, including measuring BP, dipstick urine test for proteinuria and symptoms, which includes chest pain or dyspnea, headache or visual disturbances, and vaginal bleeding with abdominal pain. When maternal risk is calculated, the POM app displays recommendations appropriate to the pregnant woman's condition, such as treatment with oral antihypertensive medication, transport to the hospital within 24 h, intramuscular magnesium sulfate injection (MgSO4) and urgent recommendation for transport to the hospital within four hours. As a part of the CLIP Pakistan trial, LHWs in rural Pakistan used the POM app during their visits to pregnant women to deliver the clinical intervention.

### Participants in Qualitative Evaluation

The LHW program is a national program launched in 1994 to improve access to primary healthcare at the community level in rural and urban slum areas of Pakistan (13). LHWs are women from the communities they serve and provide health education, basic preventative health services and family planning services at the woman's home as well as act as a liaison between the formal health system and the community (13, 14). For rural pregnant women, LHWs are their first point of contact in the health system and promote skilled care seeking during pregnancy and childbirth, educate women for danger signs and adequate nutrition as well as provide iron and folic acid supplements and tetanus vaccinations (15).

### Data Collection and Analysis

**Figure 1:**
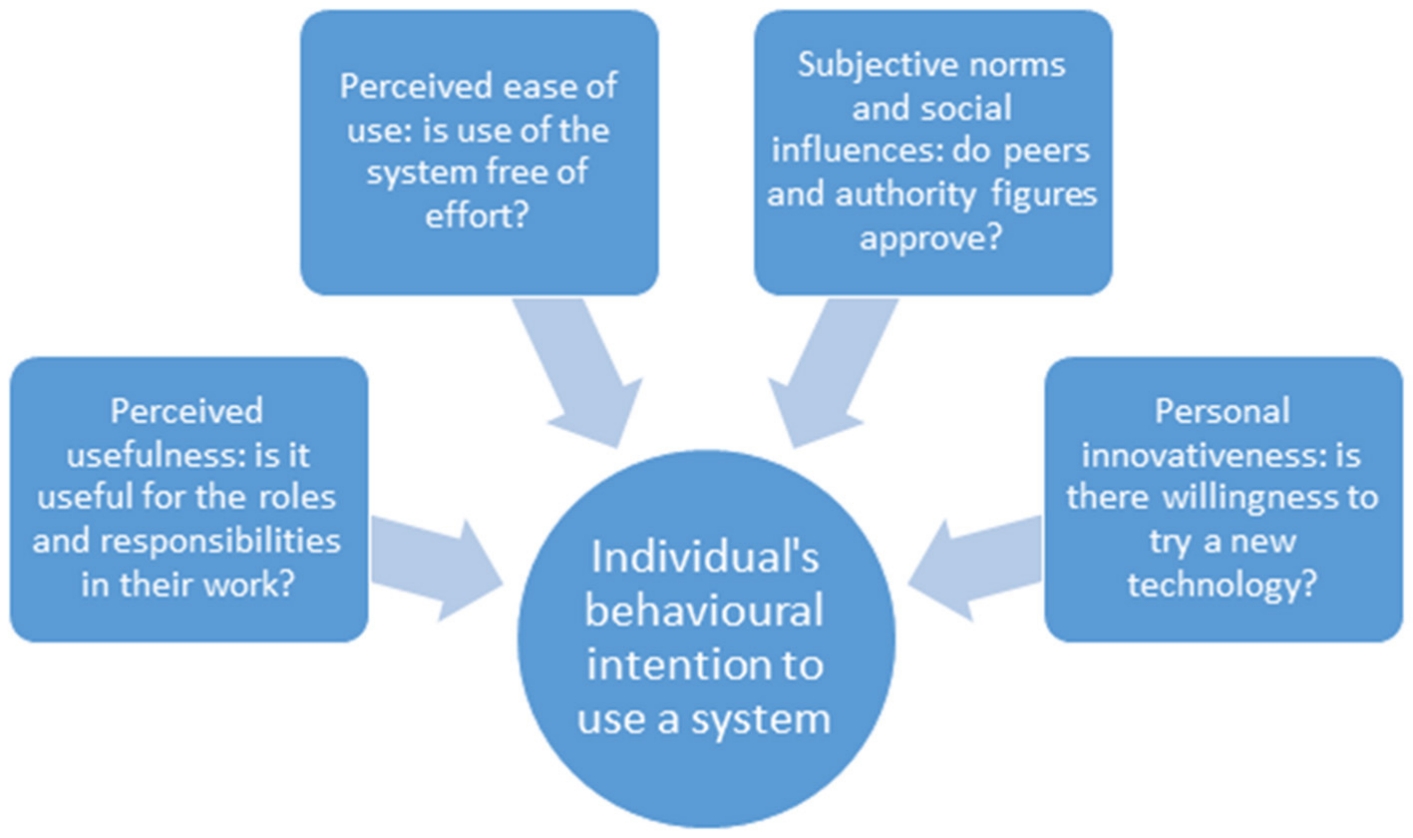
Key constructs of the Technology Acceptance Model (TAM).

The health worker evaluation was conducted between December 2017 and February 2018, around a year after the CLIP Trial ended. A semi-structured focus group guide based on the constructs included in the Technology Acceptance Model (TAM) was developed for the purpose of this study. TAM theorizes that an individual's behavioral intention to use a system is determined by perceived usefulness, defined as the extent to which a person believes that using the system will enhance his or her job performance, and perceived ease of use, defined as the extent to which a person believes that using the system will be free of effort (16). This model was expanded in relation to use of mHealth technologies to include two additional constructs: social influences or subjective norms defined as a person's perception what their informal social network or people important to him or her think about the behavior, and personal innovativeness, conceptualized as the willingness of an individual to try out any new information technology, and to highlight the importance of external barriers and facilitators to the mobile health tool (17–19) (see Figure 1).

Key informant interviews were conducted with supervisors and focus group discussions with LHWs to explore their perception of impacts on themselves, pregnant women and communities in general, and other barriers and challenges. All LHWs in the CLIP trial were eligible for the qualitative interviews. Of the total 70 LHWs across the three clusters, 16 LHWs did not participate due to time conflicts with existing commitments. A supervisor was approached and recruited from each of the three districts. With the goal of understanding perceptions on the impact of using POM, Aga Khan University researchers led designing the focus group format and guiding questions with input from University of British Columbia investigators. Interviews were framed using the SALT approach (Stimulate, Appreciate, Learn, and Transfer), a communication strategy in community engagement that aims to develop creative responses rooted in local strengths (20). Participatory reflection was employed through creating a timeline of daily activities to further understand how the POM app was utilized within LHW's lived experiences, as well as an impact diagram to explore the effects the POM app on their professional roles and in their communities. These two activities aimed to promote reflection on LHW's experiences with the CLIP intervention and the POM app in particular. At each focus group discussion, participants were broken up into smaller groups of four to eight LHWs to maximize participation in for reflection activities that were later reported to the wider group.

A small team of data collectors trained by the Aga Khan University team led focus group discussions. Data collectors were engaged as active participants in the research process. The data collectors, including facilitators and notetakers, were all women to help increase the comfort and openness of participating Lady Health Workers. Members of the Aga Khan team were also present for monitoring and quality assurance.

Focus group discussions were recorded in Sindhi and translated in verbatim first to Urdu for analysis by the Aga Khan team and then to English for analysis by the UBC team. Preliminary analysis by the Aga Khan team used the Bronfenbrenner ecological model to examine individual, relationship and community spheres of influence and interaction. Preliminary qualitative analysis by the UBC team used grounded theory to examine emergent patterns and categories were inductively developed through data analysis (21, 22). With both the Aga Khan and UBC teams, all of the interview transcripts and notes were reviewed, and emergent themes highlighted. Themes were compiled for each cluster then organized for a combined list of key themes. UBC and Aga Khan teams reviewed the preliminary list of themes to finalize a list of nodes that was coded using NVivo 12. All of the material underwent full analysis in NVivo12 using the coding framework developed (see Table 1).

**Table 1:** Coding framework.

**Theme category**	**Subthemes**
Impact of POM on care improvement	Relationships with community
	Confidence and respect in LHWs
	Pre-eclampsia and maternal health knowledge
	Community change
	Homecare
POM app features	Features of POM that LHW enjoyed and/or felt made it easy to use
	Ways to improve POM
Challenges and barriers	Implementation challenges

## Results

Three key informant interviews were conducted with two Lady Health Supervisors and one Senior Medical Officer. Sixty-two LHWs were interviewed in three focus group discussions. There was one focus group discussion in each of the three clusters. These included 30 LHWs in Tando Qaisar (81.1% of LHWs in the cluster), 17 in Moolan (100.0%), and 15 in Odero Lal Village (93.8%) (see Table 2 for cluster characteristics).

**Table 2:** Cluster characteristics.

	**Tando qaisar**	**Moolan**	**Odero lal village**
Area	81.0 km^2^	78.3 km^2^	89.8 km^2^
Population	35397	27856	33466
# LHWs	37	17	16
LHW coverage	0.97 LHW/1,000 population	0.35 LHW/1,000 population	0.24 LHW/1,000 population
Baseline neonatal mortality rate (/1,000 livebirths)	26.2	24.69	39.91

### The POM App and Impact on Lady Health Workers

#### “We Didn't Feel Any Difficulty”—Perceived Ease of Use

The LHWs interviewed in this study reported that the POM app was easy to use. An LHW said, “No, it's not difficult, very easy, I find a lot of ease in using it because it's in our own Sindhi language so it's easy and every question is easy to read.” The LHWs appreciated that it was in the local language and was on a tablet rather than pen and paper. The format on the tablet was also appreciated by the LHWs, who commented on the simplicity of check-marking danger signs and automatically appearing instructions to guide the next steps,

“We had this ability because the tab (tablet) notified disease; we only had to tick mark it. Tab used to guide us on how to proceed further; we didn't have to brainstorm for this. Tab used to guide us what we should do. If the woman's blood pressure was raised, then we had to refer that woman in four or 24 h. So that's how we used to do it with the tab's assistance.”

They liked that the app automatically checked for errors and would not continue until an incorrect entry was corrected. “It's like an advocate,” said one LHW, “It catches our every mistake. If we make a wrong entry for the question, it suggests for us to correct it. We feel good when we got the tab.” LHWs appreciated the knowledge base at their fingertips, instead of requiring the LHWs to remember all information, which they noted was what they had to do previously. As one LHW commented, “Everything has written in it! You only have to touch it (the touch screen tablet) and we find it easy.” They also liked that the POM app provided an immediate diagnosis. A LHW explained, “It means that if the pregnant woman has any trouble, we can know it through the POM device easily. If the woman has difficulty in breathing or blood pressure, we get it immediately.”

#### “We Felt Lot of Pleasure That We Gained Knowledge”—Perceived Usefulness of Increased Knowledge

The LHWs said that they found the app was useful in their work because it helped them gain knowledge about pre-eclampsia and eclampsia and associated clinical care such as measuring BP, heart rate and proteinuria, which was new to them. For example, one LHW said, “We didn't know about diseases, we couldn't check pregnant women's blood pressure, or do their urine test. But when POM device came, we got knowledge about pre-eclampsia then.” Use of the POM app was perceived to enhance their professional performance and LHWs said that the system supported them on what to say and how to talk to local women. For example, one LHW said, “The training we got it has benefitted us a lot, it has trained us how to talk and what to talk.” LHWs reported they were able to give stronger counseling because of increased knowledge and recommendations directed by the app.

#### “Now You Have Become Doctors” – Social Influences and Building Respect in LHWs

With the provisioning of tangible clinical services directed by the POM app, such as blood pressure measurements, urine tests, and administration of magnesium sulfate (MgSO_4_) injections and oral antihypertensive tablets, LHWs perceived that community members found their services more useful. LHWs felt that their work was more valued and that their respect in their communities increased, which was associated with feeling more confident in their professional roles. LHWs spoke of how they previously could only listen to the pregnant women's concerns but with the POM app, they felt empowered to be able to act. One LHW described,

“Impact is more since we got the tablet and blood pressure apparatus… by checking blood pressure, there is a lot of impact happening. Earlier, we used to go to pregnant women and listen to their troubles but couldn't do anything for them, but now when we check pressure and we got blood pressure apparatus, then it affects us a lot.”

Instead of primarily providing health education, providing services and their ability to diagnose and treat was perceived to increase the value of their visits. In their words, “Our knowledge has increased, and our respect has increased….They (community members) told us that now you people have become Doctors as well, now you know how to check blood pressure. Now you have become Doctors.” LHWs expressed joy of being consulted when earlier they were ignored. One LHW shared, “They are same women who earlier used to ignore us now they give us response. Now we feel lot of pleasure. We worked with you; and we have gained knowledge, we checked BP, and checked proteins in urine.”

### Impact on Pregnant and Postpartum Women

#### “She Got the Information While Sitting at Home”—Homecare and Reducing Barriers of Access

A key emergent theme was an appreciation of home-based visits. Because of the gender context where women may be sequestered within their home, requiring women to travel was a barrier to accessing care. Receiving counseling and care at home was reported to increase accessibility of maternal health services. LHWs from a focus group discussion said,

“There are many sick women in the community. Pregnant women also have needs and we serve them; because they cannot go outside so we serve them…. We felt a lot of pleasure that now we will check BP of pregnant women… Through this app, pregnant women's BP can be checked at home.”

In addition to homecare reducing gender barriers to accessing health care, LHWs and supervisors also discussed that it helped mitigate barriers of poverty and distance. Community members appreciated the service because it reduced costs of seeking care, which include clinic fees, costs for transport as well as opportunity costs for a male member of the family to accompany the pregnant woman. Because health care was brought into the homes of the pregnant women, it increased access for those in rural areas where the health facility was far away. A senior medical officer interviewed shared,

“It is very useful, in system, and a lot of benefit is given to the community, especially in rural areas. Our area is rural. Urban area women easily will reach to facility, but [in our community] (*name removed to protect confidentiality*), distance is faraway 40 km. So, regarding that in our area a lot of improvement has happened, so here, impact is a lot.”

While LHWs and their supervisors shared positive comments about community members appreciating the homecare provided, there is potential that women rely on the LHW visits instead of facility-based care. This issue can be seen in the following quote by an LHW: “Earlier, they used to go to the hospital for checkup. The advantage we had due to the tablet [is that] we can check their BP and do their check up steadily and they were free from the hassle of going to the hospital.” However, LHWs did not report resistance to referral recommendations when indicated by POM.

#### “We Do More Visits to Pregnant Women”—Strengthening Continuity of Care

A beneficial impact of implementing the POM app and home-based visits was the increased number of visits. LHWs reported that women used to receive a single visit during pregnancy. With the POM app, LHWs shared that they had more visits, which allowed for greater continuity of care in pregnancy. As one LHW described,

“We do more visits to pregnant woman… than before, which was less. If a pregnant woman has any more trouble… because more visits are there, we can easily understand their problems. For example, if any pregnant woman's blood pressure gets high, we deal with her with ease”

In addition to more visits antepartum, the POM app also promoted postpartum visits. A LHW said, “Normally, we make one visit to pregnant woman… but when we got the POM device, then we also made a visit after delivery.” The postpartum period is considered key to reducing maternal mortality rates globally as more than 60% of maternal deaths occur in the postpartum period (23).

### Impact on Health System and Communities

#### “We Tell Supervisor and She Is Happy With Us”—Strengthening Relationships With the Health System

LHWs shared that the POM app and participating in the trial strengthened their connection to the local health system. They perceived increased communication with supervisors and doctors at health facilities. A LHW recalled, “After checking blood pressure, we used to send a report to higher officials,” who appreciated the additional communication, “If pregnant woman has any trouble, she (LHWs) keeps communication. We tell supervisor, and she (supervisor) is happy with us.”

A local medical officer also spoke highly of the LHWs trained to use the POM app,

“They knew earlier but not as much knowledge [as] they have now. [Earlier,] they had to refer someone. Now, naturally they know it. Now, they treat [it] themselves, and that know that, what pre-eclampsia is…Those who don't use it, they don't have as much knowledge. Those who used it, they have a lot of knowledge. They are more useful; they can do treatment.”

#### “They Treat Us as Relatives”—Strengthening Relationships With the Community

LHWs spoke of strengthening relationships with pregnant women and community members through delivering care with the POM app. As one LHW commented, “Those pregnant women also realized that these LHWs are doing good for us and they value us and feel that we are part of them because we get along well with them, so our relationship gets more mature with them.” Other LHWs elaborated, “They are also attached with us like that as they are our relatives…. They treat us as relatives; they have become like a family.”

LHWs shared that closer relationship with community members helped build trust and increase women disclosing their pregnancies early. LHWs shared that previously, “Five months would have passed, no communication happened… Now, the woman herself would come and tell us that I am pregnant, check my blood pressure.”

#### “Change Has Come, a Lot of Change Has Occurred”—Community Change

LHWs revealed that their increase in knowledge was shared with their communities. LHWs reported that there was more awareness of pre-eclampsia, which was associated with earlier recognition and more immediate decision-making to seek care. As one LHW said, “If someone had high blood pressure, or develops fits, they didn't know about it earlier but now they recognize it early. They understand that if someone is having any trouble, then they rush to the hospital immediately.” As momentum was built, LHWs also recalled that communities asked for continuation of the program.

Empowerment of women was an emergent theme from LHWs. LHWs talked about women starting to take care of themselves, seeking health services, increasing value of maternal health, and linking the health of women to the health of the household and society at large. Take for example the following quote from an LHW,

“Women have started taking care of themselves. Women have become so smart…it gives us a lot of pleasure. Women go to the hospital by themselves to get their BP checked because they themselves feel that their BP is raised… the area has gained so much perceptiveness.”

The quote above suggests that women are starting to become more aware of their health issues and are starting to take care of themselves. This is further revealed in the quote below,

“Change has come, a lot of change has occurred…. We give suggestions to pregnant women that never happened before. Now, whether we go there or not, they do take care of themselves. Now, she can take care of her health. Now their knowledge has been increased about blood pressure that gets high or not.”

LHWs also shared stories of women starting to demand health services and that the investment in maternal health in the communities may have increased the profile of women's health among men as well.

“Women go to fields. They cut grass. We call them from a far distance, “Come to us, we will do your checkup”…. She gets her blood pressure checked. We tell her that her blood pressure is high and then, her husband comes and asks her “Why are you sitting here?”…then she replies to him, “I am not well. They have come and told me many things, now I know a lot. I will go to the Doctor and get myself treated by the Doctor. I will go to Doctor tomorrow and get my treatment done from Doctor. Now, I understand properly”…. It has impact on males also here.”

A Lady Health Supervisor said that working with maternal health is a part of empowering women in the communities. In her words,

“Some males are like that, who don't give any importance to any female…. If she is his own wife, her work is just cooking and taking care of children… They don't give her importance…. [However,] her health is important. If her life is there and her health is good, she can give time to home and children very well… When we tell women about its importance and make her realize that you are also a part of that society; your health is also important. Then, she realizes herself.”

### Implementation Challenges and Opportunities for Further Development

Within the context of sequestering women, concerns around POM largely seemed to be around taking pictures of pregnant mothers with the tablet and those pictures being shared with higher authorities and newspapers. As one LHW recalled, “Initially, [when] we went there, they said that she will take our photos…may be they will give photos to any newspaper… and she will spoil our women.” However, the LHWs said that this issue was resolved when there was more awareness of the project. An LHW shared,

“They (villagers) had misunderstandings that they (LHWs) will click photos of pregnant women from their mobiles, but when we counseled them that we checked some other woman to through this app and we checked heartbeat, oxygen saturation through sensors and her pictures were not clicked, then they said, ‘Oh, that's such a good thing’.”

There were some implementation challenges around timing of registration and some confusion where people thought they were viewing the baby's heartbeat with the sensor, not the mother's. Contextual community challenges included lack of transport and distance to facility, gender norms as well as poverty and malnutrition in the communities, which were associated with requesting incentives. LHWs were sympathetic and commented on the increasing poverty in the rural communities. Challenges of the LHW program that influenced implementation included situations when the LHWs were not paid for many months and overall low coverage of LHWs in the clusters (see Table 1).

LHWs also spoke of other health conditions in the communities that concerned villagers, especially a need to focus on chronic conditions like diabetes and hepatitis B and C. LHWs did not recommended changing any app features to improve usability but instead recommended that other health conditions in the communities should be added to the app. Vaccines, nutrition, and family planning were also recommended to be added to the app. Some asked for the entire LHW handbook to be put onto the app and others also recommended integrating the app within the health system. As one LHW described, “We did entry on the tablet… if we told the Doctor, he will be able to help more… we can interlink with the Doctor, we can keep in touch with the Doctor, and send the report immediately to the Doctor.”

## Discussion

An mHealth tool's ease of use is seen as critical to the perception and uptake by community health workers (12). Fitting with the concepts of TAM, LHWs reported that being in the local language, the check-mark format for danger signs, digital instead of pen-and-paper, easy to access information, simplicity of instructions that automatically appeared, automatic checking for errors, and immediate diagnosis made the app easy to use. Additionally, LHWs perceived the app was useful for their work because it provided knowledge about pre-eclampsia, eclampsia, and related clinical care as well as guided how to counsel women. LHWs also felt that communities valued their increased capacities for care, which supported LHWs' confidence to use the system and perceived usefulness of the app.

TAM posits that perceived usefulness and ease of use were influential factors in determining the intent to use a novel innovation (16). However, TAM was found to only account for about 40% of a technology system's perceived value, demonstrating a need to look into human and social change processes that also influence acceptance (24). Even with the addition of social influences and personal innovativeness, the latter of which did not appear in LHW's comments similar to Hoque's (2016) findings, TAM is based on the Theory of Reasoned Action (17) and thus focuses on individual level decision-making and intention (25). Consequently, the broader SALT and ecological approaches to gathering data strengthened this evaluation by exploring the local context and potential impacts on pregnant and postpartum women as well as the wider health system and communities. This echoes findings from two recent studies that examined the use of TAM concepts in LMICs. In Bangladesh, personal innovativeness did not significantly impact to adopt mHealth (18) and a study in rural Uganda highlighted the importance of external contextual factors and found that barriers and facilitators external to the mHealth intervention were key issues that influenced utilization (19).

Though both socioeconomic and ethnic factors play important roles in negotiating the experiences of women in Pakistan; Pakistani society is patriarchal with strict segregation of the sexes (26). Due to the institution of *purdah* that supports demarcation of male and female spaces and roles and is interlinked with *izzat*, the honor of men, women are often confined to their home (26). This was recognized by the Ministry of Health, which promoted the need for outreach and community-based services by female health workers (26). Within this context, LHWs discussed how their services were valuable and appreciated because pregnant women could receive clinical care within their home.

The expansion of the LHWs' responsibilities to more clinical care, supported by the POM app, impacted their role as community health care providers. A previous study found that there were gaps in knowledge among community health care providers regarding causes of pre-eclampsia and eclampsia and its management (27). Additionally, a study on the potential for task-sharing pre-eclampsia identification and emergency management to LHWs found that the lack of capacity to actively do clinical examinations and administer drugs were reasons that community members did not value their services (15). In this study, many LHWs spoke about how their communities valued their services more and how they became more respected as health care professionals.

Kane et al. (28) write that, “For CHWs to be able to function as health promotion practitioners and to be able to empower the citizens and communities they serve… it is essential that they themselves be and feel, empowered.” While preventative health promotion activities are at the core of CHW responsibilities in LMIC contexts (28), this research suggests that the introduction of clinical responsibilities and the provision of basic clinical care was both possible and empowering for LHWs. Their expanded role gave LHWs a sense of greater control and increased value from the perspective of their communities. This research shows that the LHWs were happy to have gained skills in measuring blood pressure and having the blood pressure device with them.

The importance of the LHW's role in their community is heightened due to decentralization of the health system in Pakistan. The federal Ministry of Health in Pakistan was disbanded in 2011 and its responsibilities devolved to the provinces to better coordinate various, previously distinct and parallel programs (14). However, Bhutta et al. (14) noted that although health was now a provincial responsibility, there was not a corresponding distribution of resources and much of the existing expenditure within the health sector is restricted to tertiary hospitals in the urban areas. LHWs are the primary health worker cadre in rural Pakistan, but support for their services and the continuum of care to referral facilities was insufficient (14). LHWs spoke of the investment in their services as a way to build better relationships with the health system and overall community. Investments in mHealth as a platform for strengthening LHW capacity has the potential to strengthen these linkages and address healthcare challenges that drive poor outcomes more broadly.

Beyond the health system, some LHWs spoke about personal empowerment within their communities. Working to reduce gender inequalities that exacerbate disparities in health is a sensitive issue. A community-level intervention requires community support; however, entry into a community and cultivating community support is often regulated by gatekeepers, who themselves already hold power and likely benefit from the existing local power structures. LHWs recalled how communities asked for continuation of the program and how their professional status increased in the villages suggests community support for the intervention. At the same time, LHWs spoke of how providing health care was a way to talk about increasing the status of women and their ability to take care of themselves. Any decision about continuation of the mHealth program will depend on local interpretation of the trial results and associated economic analyses that are currently undergoing peer review. However, we are sensitive to having empowered these health workers and then having removed the vehicle of that empowerment.

However, while LHWs and their supervisors spoke of increasing capacity of their services and respect as health care professionals in their communities and even some discussion around strengthening relationships and opening a space to explore gender empowerment, there is a concern on the eroding trust and value of LHWs with study completion. The LHWs did not comment much on the subject of how their services and roles were impacted after the closing of the project and when the app was removed because it had not proven its efficacy. However, the lack of comments on the subject may suggest a possible social desirability bias in responses when speaking to researchers from the Aga Khan University, which community health workers and members held in the highest esteem. Though focus group discussion facilitators and note-takers were hired as part of the qualitative study, LHWs had previous interactions with members of the Aga Khan team as part of the CLIP trial. While Aga Khan team members were not actively involved in the discussions to avoid potential bias, their presence may have encouraged LHWs to emphasize their appreciation of the project in the area.

Some limitations of the study include a possible social desirability bias in responses and a relatively short time-frame of the project to witness social and behavioral changes. Additionally, this study only evaluated the POM app implementation with LHWs and their immediate supervisors. Consequently, the potential impacts on pregnant and postpartum women, health system and on the wider community were filtered through the perspectives of LHWs. For example, feasibility studies found that perceived poor quality of care at public hospitals was a significant barrier to many women choosing to access health services (29). By exploring the impacts of the POM app on LHWs, issues around quality of care at facilities and other key barriers, such as poor availability of transport, financial constraints and the unavailability of chaperones (29), were inadequately captured.

## Conclusion

In conclusion, LHWs found the mHealth app easy to use and useful for their work. It was a helpful repository for maternal health information and helped guide counseling and management of pre-eclampsia. Beyond the mHealth app itself, LHWs appreciated the investment in their skills and capacity. Capacity building for LHWs may be an area of neglect, which is a critical gap considering the large burden of responsibility they hold for community and rural health. Investing in communications technology for community health workers may strengthen cohesiveness of the health system overall in improved referrals and follow up. Determining how to sustain the beneficial impacts LHWs' skill and capacity remains unresolved.

## Data Availability Statement

De-identified raw data supporting the conclusions of this article will be made available by the authors, without undue reservation.

## Ethics Statement

The studies involving human participants were reviewed and approved by The Aga Khan University (AKU, 2590-Obs-ERC-13) and the University of British Columbia (UBC, H12-03497). The patients/participants provided their written informed consent to participate in this study.

## Author Contributions

PD, ZB, RQ, LM, MV, and BP designed the CLIP trial and components of the intervention. BP, RQ, SaS, SuS, and ML designed this health worker evaluation study. SaS and SB implemented the health worker evaluation and were responsible for data collection, transfer and data cleaning. M-LK, SaS, BP, and MV performed the analyses. M-LK and BP wrote the first draft of the manuscript. All authors provided feedback and review of the manuscript.

## Conflict of Interest

The authors declare that the research was conducted in the absence of any commercial or financial relationships that could be construed as a potential conflict of interest.

## CLIP Pakistan Working Group

Amjad Hussain, Javed Memon, Farrukh Raza, Sharla K. Drebit, Chirag Kariya, Mansun Lui, Diane Sawchuck, Ugochi V. Ukah, Mai-Lei Woo Kinshella, Shafik Dharamsi, Guy A. Dumont, Tabassum Firoz, Ana Pilar Betrán, Susheela M. Engelbrecht, Veronique Filippi, William A. Grobman, Marian Knight, Ana Langer, Simon A. Lewin, Gwyneth Lewis, Craig Mitton, Nadine Schuurman, James G. Thornton, France Donnay, and Kelly Pickerill.
